# A Feasible Computational Fluid Dynamics Study for Relationships of Structural and Functional Alterations with Particle Depositions in Severe Asthmatic Lungs

**DOI:** 10.1155/2018/6564854

**Published:** 2018-07-22

**Authors:** Sanghun Choi, Shinjiro Miyawaki, Ching-Long Lin

**Affiliations:** ^1^School of Mechanical Engineering, Kyungpook National University, Daegu, Republic of Korea; ^2^Jacobs, 1100 NE Circle Blvd., Suite 300, Corvallis, Oregon 97330, USA; ^3^Department of Mechanical and Industrial Engineering, IIHR-Hydroscience and Engineering, University of Iowa, Iowa City, Iowa, USA

## Abstract

This study aims to investigate the effect of altered structures and functions in severe asthma on particle deposition by using computational fluid dynamics (CFD) models. Airway geometrical models of two healthy subjects and two severe asthmatics were reconstructed from computed tomography (CT) images. Subject-specific flow boundary conditions were obtained by image registration to account for regional functional alterations of severe asthmatics. A large eddy simulation (LES) model for transitional and turbulent flows was applied to simulate airflows, and particle transport simulations were then performed for 2.5, 5, and 10 *μ*m particles using CFD-predicted flow fields. Compared to the healthy subjects, the severe asthmatics had a smaller air-volume change in the lower lobes and a larger air-volume change in the upper lobes. Both severe asthmatics had smaller airway circularity (*Cr*), but one of them had a significant reduction of hydraulic diameter (*D*_h_). In severe asthmatics, the larger air-volume change in the upper lobes resulted in more particles in the upper lobes, especially for the small 2.5 *μ*m particles. The structural alterations measured by* Cr* and *D*_h_ were associated with a higher particle deposition. *D*_h_ was found to be the most important metric which affects the specific location of particle deposition. This study demonstrates the relationship of CT-based structural and functional alterations in severe asthma with flow and particle dynamics.

## 1. Introduction

Asthma is pathologically characterized by combined phenotypes of airflow obstruction, bronchial hyperresponsiveness, and airway inflammation [1]. However, how structural and functional alterations of asthma affect flow structure and particle deposition is yet to be investigated. In imaging studies of asthma, ventilation defects and airway structural changes have been investigated by using magnetic resonance image (MRI), positron emission tomography (PET), and single-photon emission computed tomography (SPECT) [2–4]. In addition, via quantitative computed tomography (QCT) imaging, several studies [5–8] have demonstrated significant alterations such as reduced airway diameter as well as increased wall thickness and air trapping. Although QCT can provide structural measurements of the airways up to segmental airways (~2 mm), quantification of local functional variables is still limited.

Image registration technique has been utilized to provide functional information by matching images at different inflation levels [9]. The registration derived-variables were validated by comparing ventilation maps from different imaging modalities [10]. Furthermore, this technique has shown strengths when characterizing functional alterations of diseased lungs [11, 12]. For instance, a study used the technique to differentiate airway vs. parenchymal phenotypes in a chronic obstructive pulmonary disease (COPD) [11]. We have recently demonstrated that volume changes of severe asthmatic lungs are preferentially smaller near basal regions and the smaller volume changes are compensated with air-volume change in apical regions [12]. We also demonstrated that severe asthmatics were characterized by the reduced airway diameters and noncircular shapes, unlike healthy and nonsevere asthmatics [13].

Computational fluid dynamics (CFD) technique has been used to analyze flow characteristics and particle depositions in the human lungs [14]. With regard to CFD simulations of asthmatic lungs, a study [15] showed alterations of particle depositions with an asthmatic subject before and after asthma attack, and another study [16] correlated forced expiratory volume in one second (FEV_1_) with CFD-based resistance before and after bronchodilator. However, both of them imposed parabolic velocity profiles to the trachea regions and uniform pressure boundary conditions to QCT-resolved ending branches, under the Reynolds-averaged Navier-Stokes equations (RANS) model or laminar assumption. Heenan et al. [17] and Jayaraju et al. [18] have compared CFD simulations using RANS model with experimental results, concluding that ones with RANS provided less accurate prediction in both flow structure and particle deposition. On the other hand, Longest et al. [19] and Tian et al. [20] have demonstrated that low-Reynolds number *k*-*ω* approximation with near-wall correction improves the prediction of particle deposition. With five asthmatics, Vinchurkar et al. [21] have also demonstrated a good agreement of aerosol delivery between imaging-based CFD method and in vivo experimental data. Meanwhile, Backer et al. [22] have demonstrated that lobar distributions of air-volume change between SPECT and QCT are consistent, so they have emphasized the importance of subject-specific boundary conditions in CFD simulations.

The main objective of this study is to investigate how functional and structural alterations of severe asthmatic lungs affect flow structures and particle depositions. This study further evaluates clinical applications of CFD in localizing hot spots. According to our existing population-based analysis [8, 12, 13], severe asthmatics were characterized by the shifted lobar air-volume change from lower lobes to upper lobes, decreased airway circularity (*Cr*), and reduced hydraulic diameter (*D*_h_). To reproduce regional air-volume change, we employed an image registration technique for a subject-specific boundary condition. Next, to reflect subject-specific airway features such as* Cr* and *D*_h_, we employed a surface fitting method from 1D skeleton to 3D CT-resolved airways [23]. We then performed computational fluid-particle dynamics simulations to obtain local particle distribution and deposition. To achieve main objective, three major components in this study are (1) reproducing regional air-volume change, (2) quantifying subject-specific airway features, and (3) quantifying local particle distribution and deposition.

## 2. Methods

### 2.1. Human Subjects

The imaging study and protocols for acquiring QCT images at both total lung capacity (TLC) and functional residual capacity (FRC) were approved by Institutional Review Board (IRB) of University of Pittsburgh as a part of multicenter Severe Asthma Research Program (SARP) [24]. Four human subjects were employed in this study, among whom two subjects were healthy and two subjects were severe asthmatic (Table 1). The CT images were taken via GE helical VCT-64 slice scanner with the slice thickness of 0.625 mm. Major criteria used to define severe asthma included treatments with oral corticosteroids and high-dose inhaled corticosteroids, besides several minor criteria [24]. At least, one major and two minor criteria were required to be classified as severe asthmatics. The CT scans were acquired in a supine position, and 3D airways, 1D skeletons, lungs, and lobes were segmented using Apollo software (VIDA Diagnostics, Coralville, Iowa).

### 2.2. Flow Simulation and Boundary Condition

Filtered continuity and incompressible Navier-Stokes equations read

(1a) ∇·u−=0,

(1b) ∂u−∂t+u−·∇u−=−1ρf∇p−+∇·νf+νT∇u−

where $\overset{-}{u}$, *ρ*_f_, $\overset{-}{p}$, *ν*_f_, and *ν*_T_ are filtered velocity vector, fluid density, filtered pressure, fluid kinematic viscosity, and subgrid-scale turbulent eddy viscosity [25], respectively. A large eddy simulation (LES) model was adopted to resolve laminar-transitional-turbulent flows in the central airways. The properties of *ρ*_f_ and *ν*_f_ were set to 1.12 kg/m^3^ and 1.64 × 10^-5 ^m^2^/s, respectively. A characteristic Galerkin finite element method [26] was employed to discretize (1a) and (1b). A moderate steady-inspiratory flow-rate of 3.27 × 10^-4 ^m^3^/s (≈20 liters/min) was imposed as the inlet condition, being equivalent to the peak flow-rate of a sinusoidal waveform with a tidal volume of 500 mL and a period of 4.8 s. Reynolds numbers (Re) in trachea range from 1300 to 1700 in the given flow-rate and individual tracheal sizes of two healthy and two severe asthmatics.

**Figure 1:**
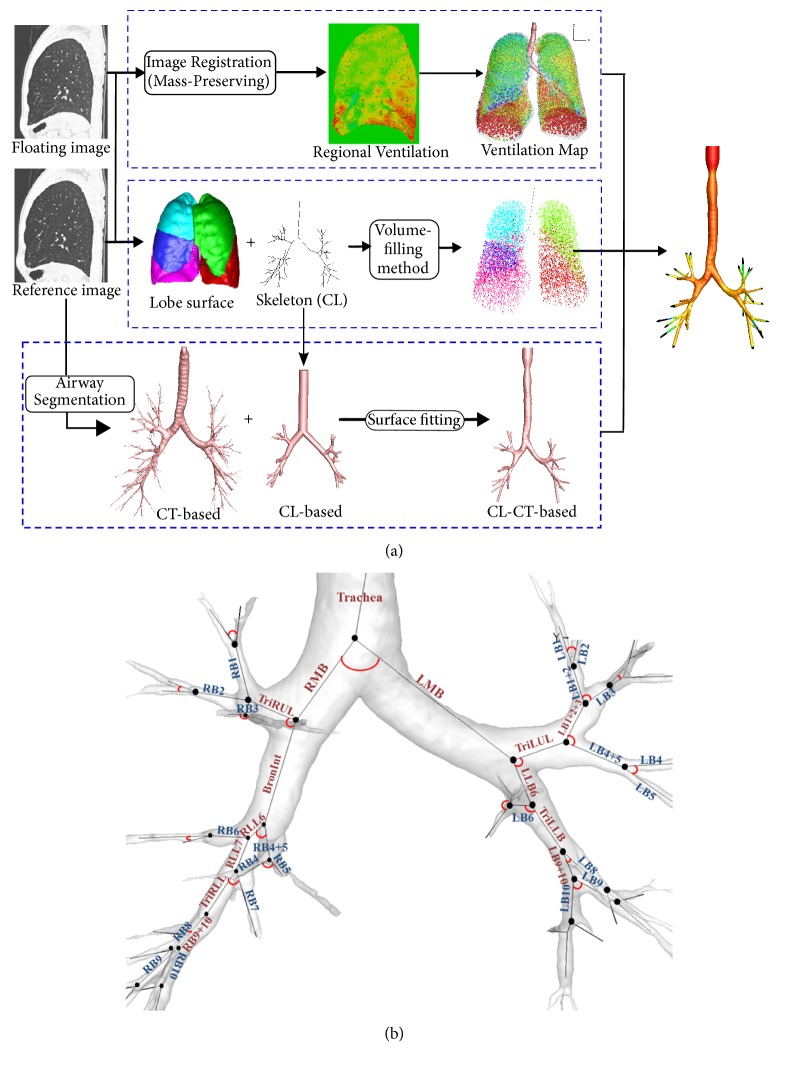
(a) Flow charts of connecting CT imaging-based structural and functional data for physiologically consistent CFD simulations. (b) Segmental names of airways: each angle of the segment represents the bifurcation angle between two daughter branches.

Figure 1(a) shows a flow chart from the image segmentation and registration to a realistic CFD simulation. First, an image registration technique [9] was employed to estimate local air-volume change between TLC and FRC at lung parenchyma (top row), and one-dimensional (1D) centerline (CL) tree structures obtained by a volume filling method [27] bridged three-dimensional- (3D-) resolved ending branches and lung parenchyma (middle row). Air-volume change measured at parenchyma was summed at CT-resolved ending branch to estimate regional flow-rate ratios. With the flow-rate ratios, parabolic profiles of velocity were essentially imposed at the CT ending branch. Lastly, a surface fitting method [23] together with Gmesh [28] was used to fit an initial CL-based 3D surface geometry to CT-segmented airway surface and construct a CL-CT-based airway geometrical model (bottom row). The resulting model captured subject-specific airway features such as* Cr* and *D*_h_. At this step, we created a synthetic turbulence [29] right above the glottal constricted regions, because the SARP study [24] did not gather oropharynx scans. The turbulent intensity and largest eddy size were set to 0.29 and 8 mm, respectively, to mimic the turbulent flows found in the CFD simulations with oropharynx [30]. The number of elements ranges from 3.9 to 5.0 million for four cases. Time steps of 5.0 × 10^−6^ s (HS 1, HS 2, and SA 1) and 3.0 × 10^−6^ s (SA 2) were chosen to satisfy Courant–Friedrichs–Lewy (CFL) number less than 1.

### 2.3. Structural Metrics Associated with Aerosol Delivery

*Cr* and *D*_h_ could be directly associated with airflows and particle delivery. In this study, we measured the structural metrics at 31 segmental airways (Figure 1(b)).* Cr* was computed to assess the degree of elliptical shape of an airway cross section as follows:

(2) $$\begin{array}{c} Cr=\frac{\text{Perimeter of an area-equivalent circle}}{\text{Perimeter of a luminal area}}=\frac{\pi D_{ave}}{P_{e}}, \end{array}$$

where *D*_ave_ and *P*_e_ are average diameter and perimeter of an airway. *D*_ave_ was computed as follows: $\sqrt{4\times A_{c}/\pi }$, where *A*_c_ is the cross-sectional area of the airway. Next, in order to assess a degree of airway narrowing, *D*_h_ was computed as follows:

(3) $$\begin{array}{c} D_{h}=\frac{4A_{c}}{P_{e}}. \end{array}$$

Note that* Cr* decreases as airway lumen becomes elliptic, and *D*_h_ decreases as airway narrowing is dominant.* Cr *and *D*_h_ were decreased in severe asthmatics compared with healthy subjects, in a population-based comparison [13]. In this study,* Cr *decreases in both severe asthmatics (SA 1 and SA 2), but *D*_h_ only decreases in a severe asthmatic subject (SA 2). In Results, the effects of* Cr* and *D*_h_ on particle delivery are investigated.

### 2.4. Particle Simulation

To compare the characteristics of particle distribution and deposition such as pharmaceutical aerosols between healthy subjects and severe asthmatics, particle transport analysis was conducted using LES-predicted airflow fields. Quasi-steady airflow fields for 2.4 s were collected, e.g., 400 datasets × 0.006 s, after initial 2.4 s airflow fields. For particle simulation, Lagrangian particle tracking algorithm [31] was adopted to track particle trajectories as follows:

(4) $$\begin{array}{c} \frac{du_{p}}{dt}=\frac{U}{Stk\cdot D_{ave}}\left(\;\overset{-}{u}-u_{p}\;\right)+\frac{\rho_{p}-\rho_{f}}{\rho_{p}}g, \end{array}$$

where **u**_p_, *ρ*_p_, and **g** are particle velocity, particle density, and gravitational acceleration, respectively. In the equation, Stokes number (Stk) is defined as follows:

(5) $$\begin{array}{c} Stk=\frac{2Q\rho_{p}d^{2}}{9\pi \mu_{f}{D_{ave}}^{3}}C_{c}\alpha^{3.7} \end{array}$$

where* Q*,* d*, *μ*_f_, *C*_c_, and *α* are the flow-rate of the branch, the diameter of particles, fluid dynamic viscosity, the Cunningham slip correction factor, and the particle-particle interaction factor, respectively. The detailed descriptions of particle transport simulation could be found in [30]. At the beginning of particle simulation, the spherical particles were uniformly distributed in a cylinder with a radius of 10 mm and a depth of 4 mm at the trachea inlet. They were then released 9 different times to obtain an ensemble average of particle deposition. The number of particles was set to 10,000, and three different spherical particle sizes of 2.5, 5, and 10 *μ*m were chosen in this study. The aerosol size was chosen with a general distribution of aerosols using dry powder inhaler (DPI) and soft mist inhaler (SMI) [32]. This is to investigate the relationship between aerosol size and airway structure. The particle density and mean free path were given as 1000 kg/m^3^ and 68 nm, respectively. “Particle distribution”, “deposition”, and “advection” by lobe are defined as “particles entering each lobe”, “those deposited in 3D segments of each lobe”, and “those exiting 3D ending branches of each lobe without being deposited in 3D segments”, respectively.

## 3. Results

### 3.1. Pulmonary Function Test (PFT)

Table 1 shows demographic as well as PFT- and CT-based measurements. Bronchodilator was performed for both healthy subjects and severe asthmatics to obtain maximal lung functions, and CT scans were acquired after bronchodilator because the aim of SARP study was to assess lung function of stable asthma [33]. First, the baseline and maximal FEV_1_ and FVC %predicted values in two healthy subjects were close to normal ranges (~100% predicted). In contrast, baseline FEV_1_ % predicted values of SA 1 (34%) and SA 2 (40%) were much smaller than normal ranges (≥80%). The maximal FEV_1_% predicted value (~46%) of SA 2 was still lower even with bronchodilator. In the same subject, the maximal FVC % predicted value was very close to normal ranges, leading to a significantly reduced FEV_1_/FVC (38%). Thus, the baseline FEV_1_% predicted values of both SA 1 and SA 2 were small values, but the lung function of SA 1 (38%↑) is relatively reversible than that of SA 2 (6%↑). According to baseline and maximal PFTs, SA 2 is likely to have significant airway narrowing despite bronchodilator, whereas airways of SA 1 might have dilated with the aid of bronchodilator.

### 3.2. CT-Based Structural and Functional Characteristics

**Figure 2:**
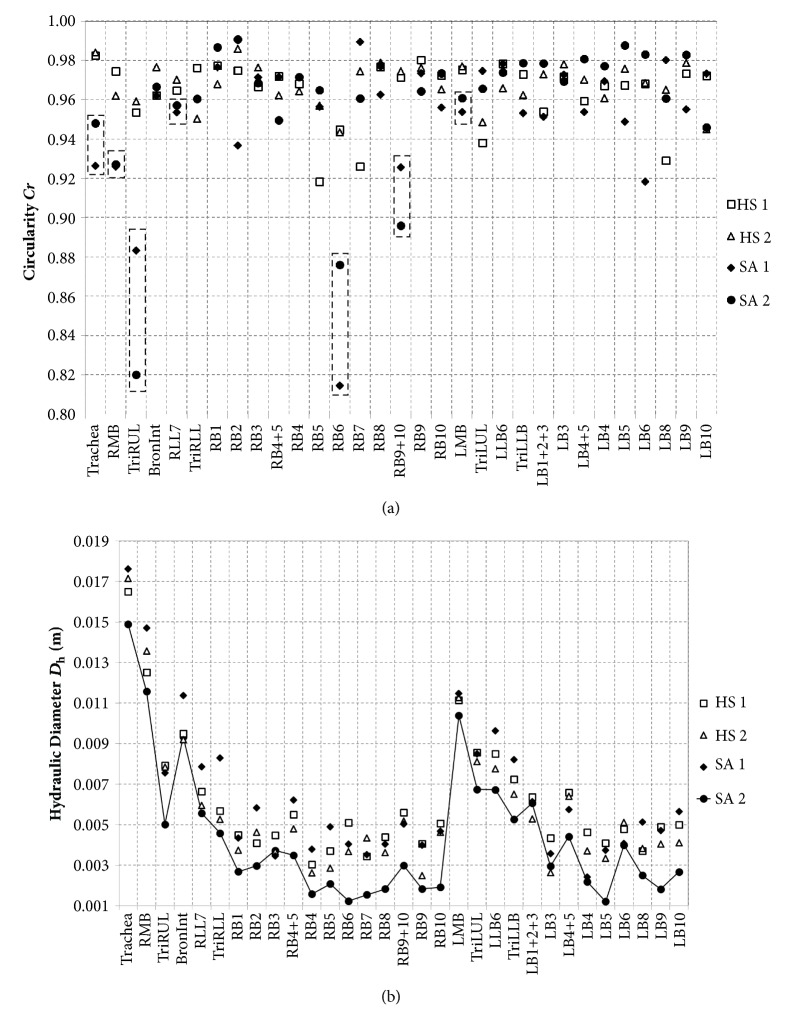
Structural variability of (a)* Cr* and (b) *D*_h_ for 31 segmental airways after 1st generation in two healthy subjects and two severe asthmatics.

**Figure 3:**
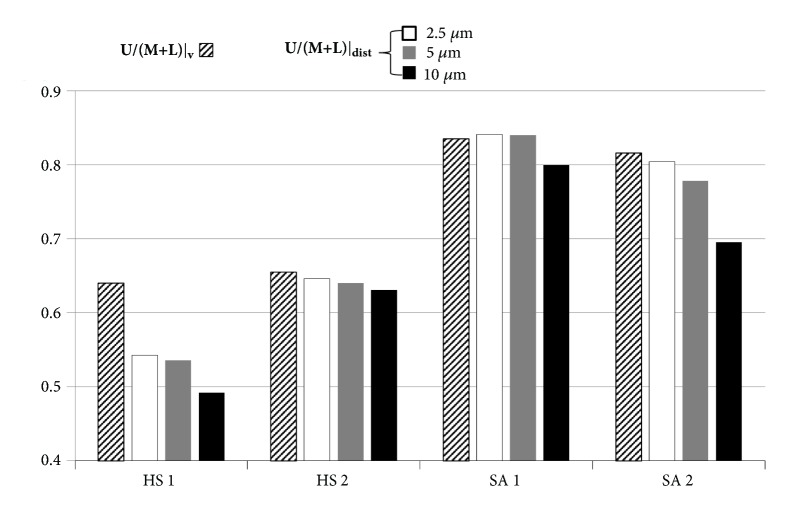
U/(M + L)|v (the ratio of air-volume change in upper lobes to middle and lower lobes) and U/(M + L)|_dist_ (the distribution ratio of particles in upper lobes to middle and lower lobes) according to the particle sizes.

We compared structural quantities of* Cr* and *D*_h_ in two healthy subjects and two severe asthmatics (Figure 2).  Figure 2(a) shows that* Cr* of two severe asthmatics was reduced compared to those of two healthy subjects. For example,* Cr* of Trachea, RMB, TriRUL, RLL7, RB6, RB9+10, and LMB in two severe asthmatics were deviated from those of the same airway segments in healthy subjects. Both SA 1 and SA 2 had the prominently reduced* Cr*, but only SA 2 demonstrated significantly smaller *D*_h_ compared with HS 1, HS 2, and SA 1 in CT-resolved airways (Figure 2(b)), which was also reflected in PFT measurements at postbronchodilator. The effect of reduced* Cr* in RMB and TriRUL is assessed between “HS 1, HS 2” and “SA 1, SA 2”. Next, the effect of constricted branch on particle deposition is mainly assessed with SA 2. In addition, SA 1 and SA 2 were characterized by reduced air-volume change in lower lungs along with elevated air-volume change in upper lungs, as compared with the healthy subjects, being quantified by the air-volume change in upper lobes to air-volume change in middle and lower lobes, i.e., U/(M + L)|v (Figure 3).

### 3.3. Regional Volume Change vs. Particle Distribution

Given the regional air-volume change, particle distribution was evaluated with three different particle sizes (Figure 3). With 2.5 *μ*m particles, the U/(M + L)|_dist_ (the particle distribution ratio of upper lobes to middle and lower lobes) was close to the ratio of air-volume changes (U/(M + L)|_v_), because small particles are likely to follow airflow streamlines. Next, U/(M + L)|_dist_ decreased as particle size increases due in part to the inertial effect that particles can easily move along with the flow to the lower lobes. Overall, SA 1 and SA 2 had an increased particle distribution to upper lobes compared with HS 1 and HS 2, being consistent with the alteration of air-volume change distribution in severe asthmatics. Furthermore, the particle distribution to upper lobes in severe asthmatics became more evident with decreasing particle size.

### 3.4. Circularity and Particle Deposition

**Figure 4:**
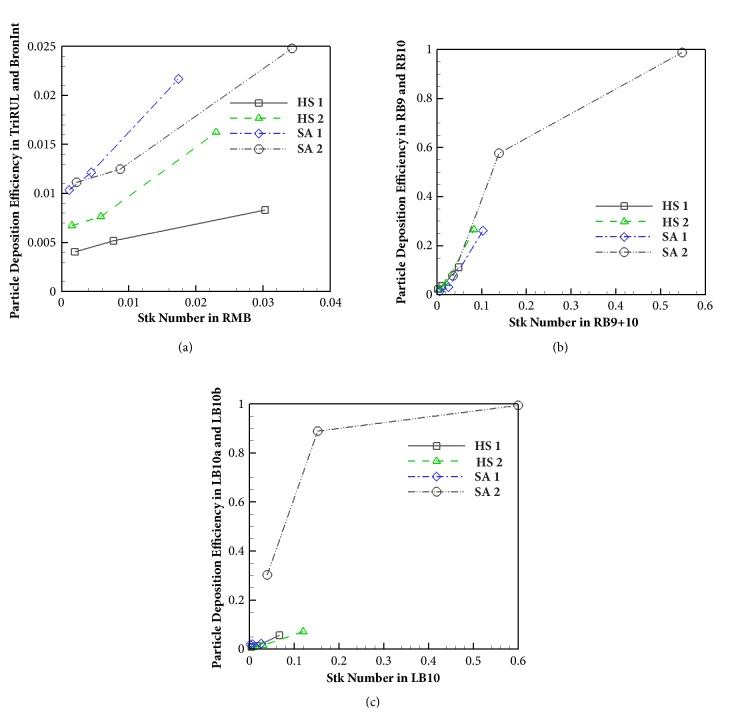
Particle deposition efficiency in (a) TriRUL based on Stk in RMB, (b) RB9 and RB10 based on Stk in RB9+10, and (c) LB10a and LB10b based on Stk in LB10.

**Figure 5:**
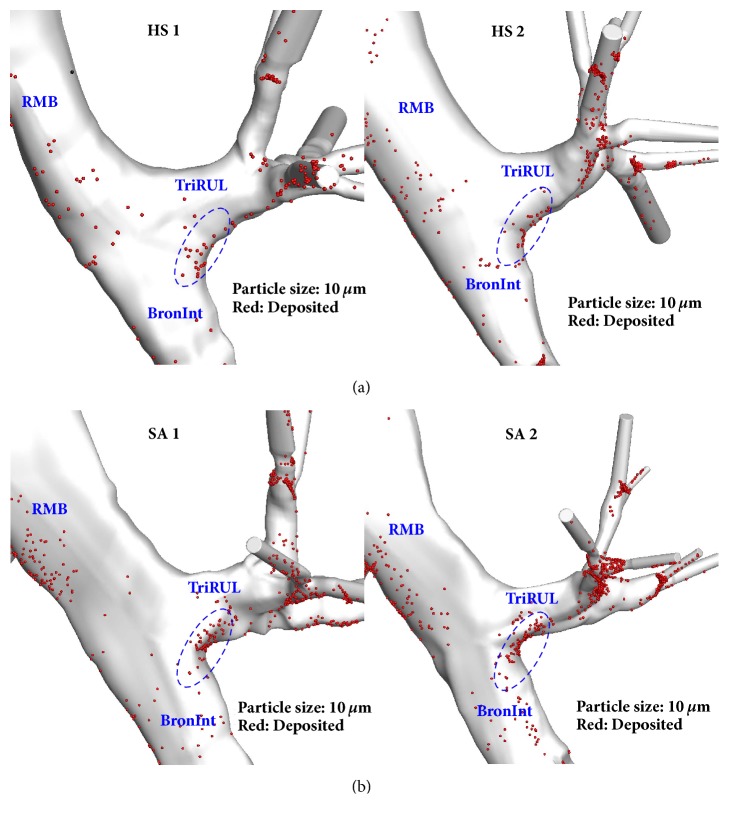
Relationships among noncircularity and particle deposition in RMB, TriRUL, and BronInt regions between (a) healthy subjects and (b) severe asthmatics. All of the images are plotted as backside-view.

Around RMB and TriRUL regions (Figure 1(b)), we compared particle depositions of two healthy subjects without elliptic shapes vs. two severe asthmatics with elliptic shapes, because SA 1 and SA 2 exhibit significantly decreased* Cr* in both RMB and TriRUL (Table 2). The two selected severe asthmatics (83° and 88°) have similar bifurcation angles as two healthy subjects (90° and 91°) at RMB, expecting similar particle depositions given the same range of Stk numbers. In Table 2, *D*_ave_ of TriRUL in SA 1 is the largest among four subjects, but *D*_h_ was slightly smaller than those of both HS 1 and HS 2 due to the reduced* Cr*.  Figure 4(a) shows the increased particle deposition of SA 1 and SA 2 in TriRUL, as compared with HS 1 and HS 2 in the same Stk range of the parent branch (RMB). Note that the comparisons based on the same Stk numbers were made to control the effects of flow-rate and airway narrowing.  Figure 5 also displays the distributions of particle deposition in TriRUL. Thus, an increase of particle deposition in both severe asthmatics was possibly due to the decreased airway* Cr*.

### 3.5. Constriction vs. Particle Deposition

**Figure 6:**
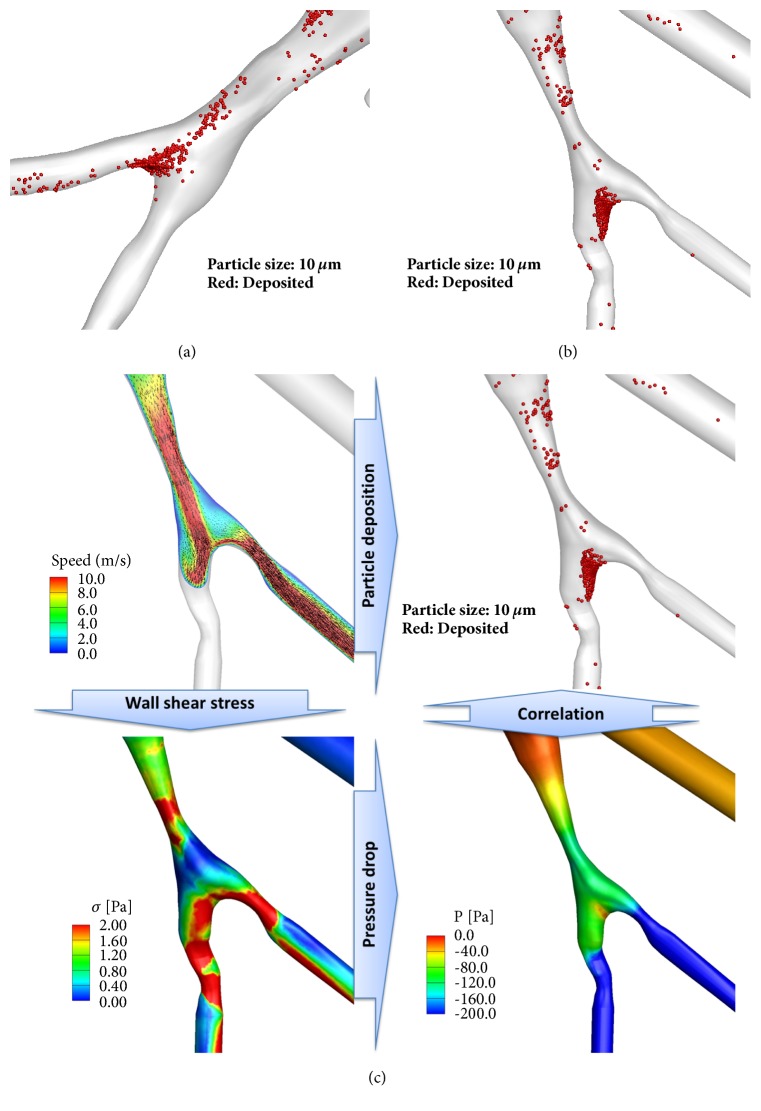
Two representative constricted regions of (a) RB9+10 and (b) LB10 in lower lobes in SA 2 subject and (c) correlations among airway constriction, wall shear stress, pressure drop, and particle deposition.

Particle deposition efficiency of the branches based on Stk numbers, daughter branches of RB9+10 and LB10, were plotted in Figures 4(b) and 4(c), respectively.  Figure 6(a) (around RB9+10) and Figure 6(b) (around LB10) show particle depositions of SA 2 with narrowed and elliptic airways. Compared with HS 1, HS 2, and SA 1, Stk is increased for SA 2 due to airway narrowing, because Stk is proportional to 1/*D*_ave_^3^ with a given flow-rate (see (5)). In the case of 5 *μ*m particles, particle deposition efficiency reached 60% and 90% for RB9+10 and LB10, respectively. For 10 *μ*m particles, particle deposition reached 100%; thus large particles could not ventilate beyond these airways. These results imply that particle deposition would be very sensitive to the reduced airway diameters due to airway constriction (see Figures 6(a) and 6(b)). We further investigated flow structure, wall shear stress, and pressure drop around LB10 (Figure 6(c)). In this airway, constriction-induced jet-flow, higher wall shear stress and large pressure drop were simultaneously observed.

## 4. Discussion

Our previous studies [8, 12] have demonstrated that air-volume change is smaller in lower lobes and larger in the upper lobes in severe asthmatics, as compared with healthy subjects. In addition, severe asthmatics were structurally characterized by smaller* Cr* and *D*_h_. In this study, we focused on the effect of structural and functional alterations in severe asthmatics on particle depositions under a moderate inspiratory flow condition (~20 liters/min). The two severe asthmatics had altered lung function characterized by larger U/(M + L)|v than the two healthy subjects. Both SA 1 and SA 2 had small FEV_1_% predicted values in the baseline lung function and showed smaller* Cr*, whereas only SA 2 subject had small FEV_1_% predicted in the maximal lung function and showed smaller *D*_h_ in CT-resolved regions. This observation implied that bronchodilator may help recover narrowed airways, but it may not recover noncircular airway shape. This emphasizes the importance of investigating airway shape besides airway diameter in particle delivery. In this study, SA 1 and SA 2 were used to evaluate the effect of* Cr *on particle deposition, and SA 2 was used to evaluate the effect of *D*_h_ on particle deposition.

First, investigating characteristics of particle deposition in severe asthmatics is important for inhaled pharmaceutical drugs [21, 34], airborne bacteria, or air pollutants. Lobar distribution of small particles was consistent with air-volume distribution (Figure 3). With increasing particle size, the delivery of particles to lower lobes may increase because of an increased inertial effect of large particles. When targeting subject-specific treatments of aerosols, understanding the mechanism of particle distribution would be critical. For example, if lower lobar bronchi need be targeted for some specific subjects, relatively larger aerosol should be treated. However, one should also consider sizes of targeted branches, because larger aerosols could not be delivered to small airways with higher generation.

We previously found that in severe asthmatics* Cr* of RMB is significantly smaller [13], so we investigated the effect of smaller* Cr* on particle deposition. With the same Stk and similar bifurcation angle of RMB, particle depositions of SA 1 and SA 2 were greater in TriRUL than healthy subjects, which could be attributed to smaller* Cr*. Airway structures with smaller* Cr* might increase the sensitivity of bacterial inflammation due to particle deposition, which would potentially increase airway wall thickness. While both SA 1 and SA 2 demonstrated smaller* Cr*, only SA 2 had constricted airways, especially in lower lobes. Particle deposition efficiency is the function of 1/*D*_ave_^3^ based on Stk numbers, given flow-rate. In other words, airway constriction could be the most important structural characteristic in association with increased particle deposition efficiency. Thus, if airways are chronically constricted in a subject, the subject may have an increased exposure of pollutants, so further reducing airway diameters along with airway inflammation. Mechanisms among airway constriction, wall shear stress, pressure drop, and particle deposition may be strongly coupled as described in Figure 6(c). First, constriction-induced high velocity creates high wall shear stress in association with high velocity gradient on the wall. A large pressure drop is required to overcome large wall shear stress. In addition, the constriction-induced high velocity might lead to an increase of particle deposition due to particle impaction in both constricted parent and daughter branches (Figures 6(a) and 6(b)). As a result, both high velocity and small *D*_ave_ may contribute to be a large Stk (see (5)), leading to an increase of particle deposition.

In this study, we only investigated two healthy subjects and two severe asthmatics, which could prevent statistical reliability, so a CFD-based population study is necessary. However, performing many CFD simulations to obtain normal distribution is computationally expensive. We have recently performed CT imaging-based clustering analysis and found clinically meaningful subgroups [35]. Since the clustering membership employed airway structure and lung function, each cluster has similar airway structure and lung function. We believe that such an approach using clustering analysis possibly reduces the number of samples by transitioning an interest from intersubject study to intercluster study. In this study, we imposed the same flow-rate on trachea inlet, due to a lack of subject-specific flow-rate information. If the subject-specific flow-rate is measured, we would further improve the comparison between healthy subjects and severe asthmatics. Furthermore, we have recently investigated airway resistance with symmetric branching angles up to the 20th generation for inspiration and expiration [36]. Then an empirical airway resistance model was introduced to estimate pressure drop due to kinetic energy and viscous dissipation. However, we have not discussed airway resistance in this study, because the number of airways was limited to investigate airway resistance. In the future, airway resistance study of severe asthmatics with realistic multiscale airways would be desirable.

In conclusion, we applied a high-fidelity CFD model together with CT image-based airway models to study particle deposition in both healthy and severe asthmatic lungs. With the aid of image registration technique, the subject-specific physiologically realistic-flow boundary condition is derived based on air-volume difference between two CT lung images of the same human subject. As expected from flow-rate distribution, particle distribution to upper lobes was larger in severe asthmatics, relative to healthy subjects. This phenomenon was more prominent when using smaller particles. In both healthy subjects and severe asthmatics, with increasing particle size, particles are distributed more toward lower lobar regions due to inertial effects. Alterations of airway* Cr* and *D*_h_ were found to be associated with particle deposition. With the same Stk, reduced* Cr *increases particle deposition. On the other hand, reduced *D*_h_ significantly elevates Stk, resulting in greater deposition efficiency. The constricted airways contribute to high wall shear stress, elevated pressure drop, and significantly increased particle deposition. It is concluded that U/(M + L)|v,* Cr, *and *D*_h_ shall be carefully considered to target subject-specific aerosols.

## Figures and Tables

**Table 1:** Demographic, PFT baseline (prebronchodilator), and maximal (postbronchodilator) lung functions and CT-based air-volumes of two healthy subjects and two severe asthmatics.

	Healthy subjects (HS)		Severe asthmatics (SA)	
	HS 1	HS 2	SA 1	SA 2
Demographics				

Gender (F, Female)	F	F	F	F

Age (yrs.)	59	29	61	48

BMI (kg/m^2^)	23.7	22.2	32.4	23.9

Asthma duration (yrs.)	-	-	10.6	19.7

Baseline lung function (pre-bronchodilator)				

FEV_1_ (liters) (% predicted)	2.97 (100%)	3.15 (94%)	1.19 (34%)	1.02 (40%)

FVC (liters) (% predicted)	3.82 (100%)	4.00 (101%)	1.83 (39%)	2.52 (80%)

FEV1/FVC (%)	78%	79%	65%	41%

Maximal lung function (post-bronchodilator)				

FEV_1_ (liters) (% predicted)	3.36 (107%)	3.17 (100%)	2.05 (72%)	1.17 (46%)

FVC (liters) (% predicted)	4.04 (103%)	3.94 (102%)	2.97 (74%)	3.11 (98%)

FEV1/FVC (%)	83%	80%	69%	38%

CT-based air-volumes				

TLC (liters)	5.37	4.31	5.64	4.33

FRC (liters)	2.04	1.79	2.85	1.77

All subjects are Caucasians and nonsmokers. PFT and CT measurements were obtained in upright and supine positions, respectively. CT scans were performed after bronchodilator.

**Table 2:** Circularity *Cr*, *D*_ave_, and *D*_h_ of two healthy subjects (HS 1 and HS 2) and two severe asthmatics (SA 1 and SA 2) in RMB and TriRUL regions (see Figure 1(b)).

	Healthy subjects		Severe asthmatics	
	HS 1	HS 2	SA 1	SA 2
RMB				
*Cr*	0.974	0.962	0.926	0.927
*D*_ave_ (mm)	12.8	14.1	15.9	12.5
*D*_h_ (mm)	12.5	13.6	14.7	11.6
TriRUL				
*Cr*	0.953	0.959	0.883	0.820
*D*_ave_ (mm)	8.3	8.2	8.5	6.1
*D*_h_ (mm)	7.9	7.8	7.6	5.0
